# Small- and medium-enterprises bankruptcy dataset

**DOI:** 10.1016/j.dib.2019.104360

**Published:** 2019-08-06

**Authors:** Peter Drotár, Peter Gnip, Martin Zoričak, Vladimír Gazda

**Affiliations:** Technical University of Košice, Slovakia

**Keywords:** Financial ratios, SME, Bankruptcy, Imbalanced data, Machine learning

## Abstract

Bankruptcy prediction is a long-standing issue that receives significant attention of academic researchers and industry practitioners. Most of the papers on bankruptcy prediction focus on companies that are listed on the stock market, and there are only limited data for the rest of the companies. These companies, not indexed at any stock market, represent a significant part of the economy. The presented dataset consists of financial ratios of Slovak companies. There are 21 distinctive financial ratios which are available for three consecutive years prior to evaluation year in which companies may have filed for bankruptcy or not. The companies come from four different industries - agriculture, construction, manufacture, retail. We provide data for four consecutive years 2013–2016 for each industry. All companies are categorized as small-medium enterprises according to EU classification. Prediction performance results on this dataset are published in the research paper “Bankruptcy prediction for small- and medium-sized companies using severely imbalanced datasets” (Zoričák et al., 2019).

**Table:** Specifications Table

Subject area	*Economics*
More specific subject area	*Financial ratios of bankruptcy prediction data*
Type of data	*Tables*
How data was acquired	*Calculated from publicly accessible records*
Data format	*Raw and analyzed*
Experimental factors	*Data were divided into four categories – agriculture, construction, manufacture, and retail* *All four categories were split into bankrupt and non-bankrupt companies* *For all industries are provided four years in which are data evaluated*
Experimental features	*Machine learning methods were used to identify bankrupt companies*
Data source location	*Annual reports of SlovakSME‘s*
Data accessibility	*Stored on Data Mendeley*[2]
Related research article	*Zoričák, M., Gnip, P., Drotár, P., & Gazda, V.* (2019)*. Bankruptcy prediction for small-and medium-sized companies using severely imbalanced datasets. Economic Modelling.* doi: 10.1016/j.econmod.2019.04.003[1].

**Table:** 

**Value of the data** • The dataset provides financial ratios of an exhaustive set of companies in four sectors: agriculture, construction, manufacture, and retail • The data can be used to propose or to benchmark statistical models or machine learning algorithms for bankruptcy prediction • The dataset can be used to investigate markers of upcoming bankruptcy • The data can be used to benchmark and validate methods for imbalanced learning, since distribution of the bankrupt and non-bankrupt companies is strongly imbalanced • Financial ratios of companies are provided for three years prior to the year when the company is evaluated as bankrupt or non-bankrupt.

## 1 Data

The dataset is accessible on Data Mendeley [2] and provides financial ratios of limited liability companies. There are three possible views on the data as depicted in Table 1:

1) Companies are divided into four different industries: agriculture, construction, manufacture, and retail,
2) Four different evaluation years are considered: 2013, 2014, 2015 and 2016
3) Two classes are defined (bankrupt (B) and non-bankrupt (NB)) for each evaluation year and industry

**Table 1:** Number of bankrupt and non-bankrupt companies per industry and evaluation year.

	2013		2014		2015		2016	
	B	NB	B	NB	B	NB	B	NB
Agriculture	1251	6	1327	6	1464	8	1652	8
Construction	1205	25	1418	30	1749	20	2174	14
Manufacture	4077	30	4450	30	5019	26	5840	14
Retail	3739	12	4404	11	5314	7	6073	4

Each company is characterized by 21 financial ratios listed in Table 2. These are provided for three consecutive years prior to the evaluation year.

**Table 2:** List of financial ratios with abbreviations.

Category	Financial ratio	Abbreviation
Activity	Total Asset Turnover	(TAT)
	Asset Turnover Days	(ATD)
	Days Total Receivables Outstanding	(DTR)
	Inventory Turnover Days	(ITD)
Liquidity	Cash Ratio	(L1)
	Quick Ratio	(L2)
	Current Ratio	(L3)
Profitability	Return on Assets	(ROA)
	Return on Equity	(ROE)
	Return on Sales	(ROS)
	Return on Investment	(ROI)
	Labor-to-Revenue Ratio	(LRR)
	Wages to Added Value Ratio	(WAR)
	Labor Productivity	(LP)
Solvency	Debt-to-Assets Ratio	(DA)
	Debt-to-Equity Ratio	(DE)
	Financial Leverage	(FL)
	Debt to Income Ratio	(DIR)
	Debt Service Coverage Ratio	(DCR)
	Asset Coverage Ratio	(ACR)
	Bank Liabilities to Debt Ratio	(BL)

**Fig. 1:**
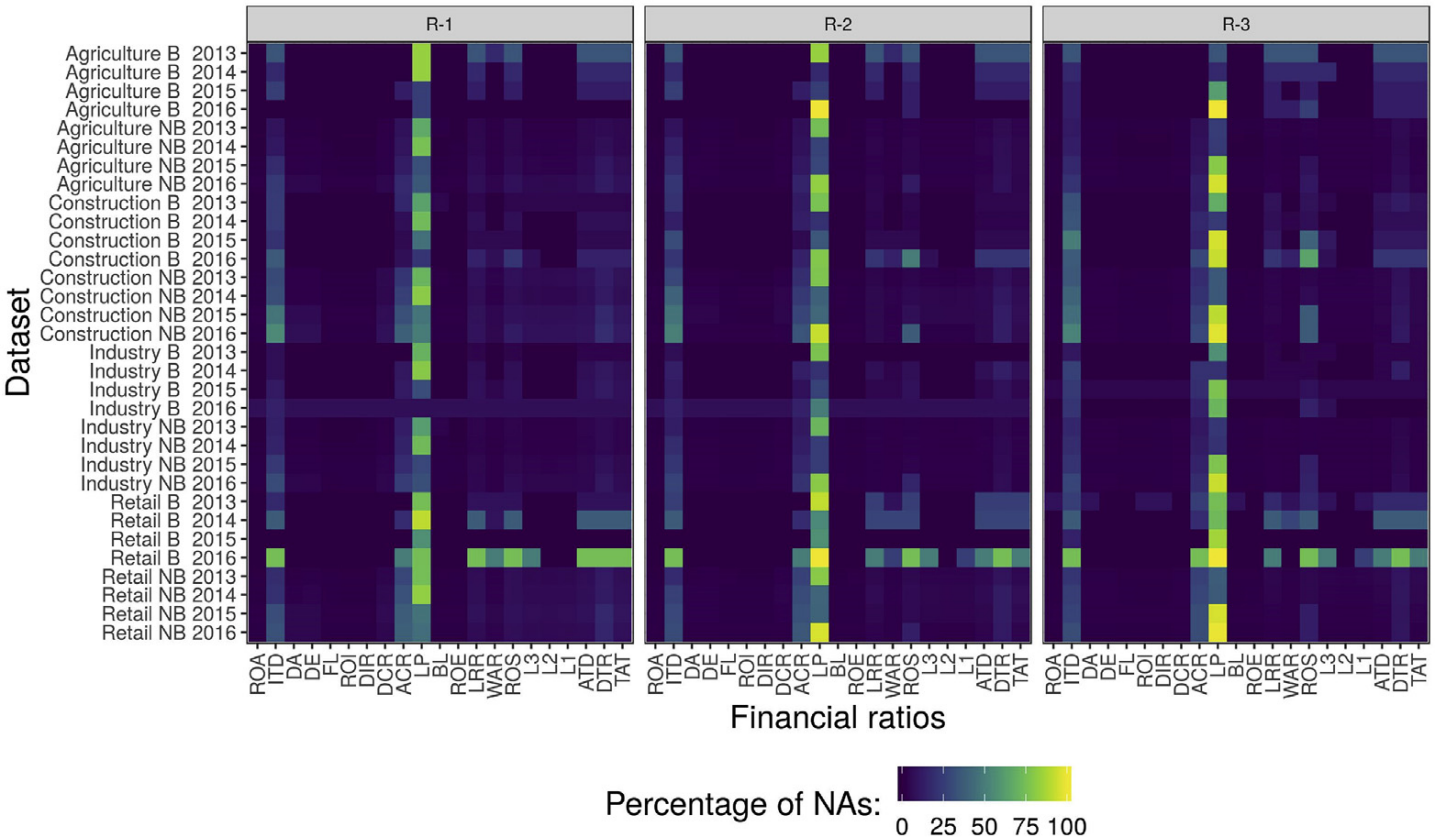
Percentage of NAs for individual datasets.

The distribution of missing values is visualized in a heatmap in the Fig. 1. Missing values for individual datasets are displayed one, two and three years prior to evaluation. The most missing values are for the financial variable Labor Productivity (LP). From the industry perspective, the most missing values are for retail with the year of evaluation 2016.

**Fig. 2:**
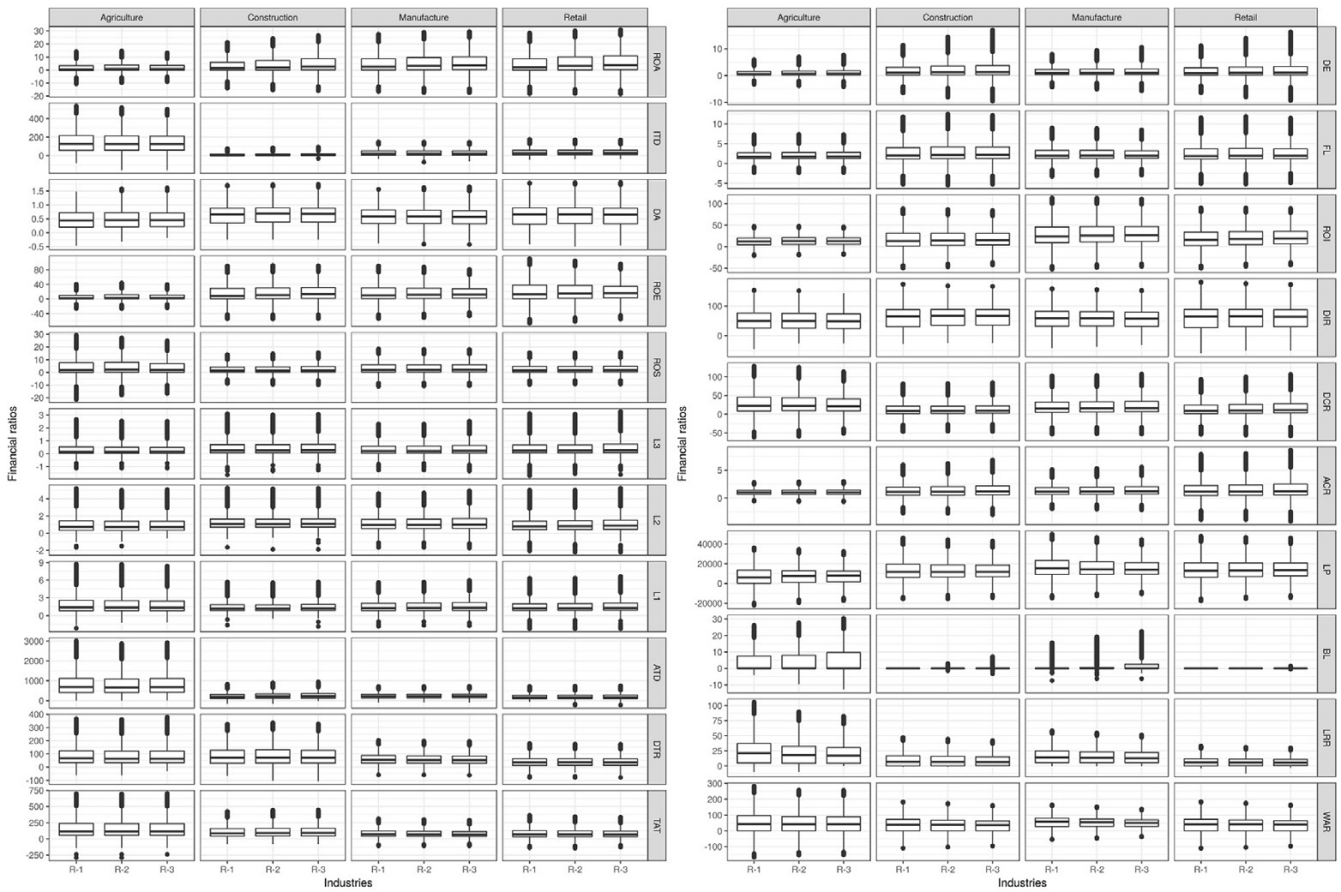
Financial ratios per industry for three years prior evaluation.

In order to provide an overview of individual variables, we provide descriptive statistics in the form of boxplots in Fig. 2. All variables include outliers for almost all years. The interquartile range is relatively stable for all variables for all industries.

## 2 Experimental design, materials, and methods

We extracted values from the financial statements of each company for all available years. Financial statements consist of balance sheet and the income statement. A balance sheet provides detailed information regarding assets, equity, and liabilities. The income statement covers revenues, costs, and profit/loss for a given accounting period. Financial statements are publicly accessible on the Register of Financial Statements [3], which is database of financial statements of all business entities operated by Ministry of Finance of Slovak Republic. We used extracted values to calculate the financial ratios listed in Table 1 using Equation (1), (2), (3), (4), (5), (6), (7), (8), (9), (10), (11), (12), (13), (14), (15), (16), (17), (18), (19), (20), (21). Based on the available data, we identified four evaluation years – 2013, 2014, 2015 and 2016. Companies were evaluated and divided into two categories: bankrupt and non-bankrupt. Companies were evaluated based on [4] with two distinctive proceedings defined for companies in financial difficulties. It is either bankruptcy procedure or restructuring. A company which begins the restructuring process may recover its financial health but, nevertheless, poses risk for its creditors. Thus, we classify companies in both the bankruptcy procedure and restructuring process as bankrupt. After classification, we selected only companies with available data for three years prior to the evaluation year. For example, for all companies evaluated in the year 2013 financial ratios are available for years 2012 (R-1), 2011 (R-2), and 2010 (R-3). There are four domains in which the investigated companies operate: agriculture, construction, manufacture and retail. Companies were included in mentioned categories based on their prevailing activity. This information is included in the balance sheet in the form of SK NACE classification.

(1) $$TAT=\;\frac{Total\;Sales}{Assets}$$

(2) $$ATD=\;365\;\frac{Assets}{Total\;Sales}$$

(3) $$DTR=365\frac{Long-and\;Short-term\;Recievables}{Total\;Sales}$$

(4) $$ITD=\;365\;\frac{Inventory}{Cost\;of\;marchandise\;sold}$$

(5) $$L1=\;\frac{Financial\;accounts}{L_{\text{denominator}}}$$

L_denominator_ = Short-term Liabilities + Short-term Financial Assistance + Current Bank Loans + Accruals

(6) $$L2=\;\frac{Financial\;Accounts+Short-term\;receivables+Acruals}{L_{\text{denominator}}}$$

(7) $$L3=\;\frac{Financial\;Accounts+Short-term\;receivables+Acruals+Inventory}{L_{denominator}}$$

(8) $$ROA=\;\frac{Net\;Profit}{Assets}$$

(9) $$ROE=\;\;\frac{Net\;Profit}{Equity}$$

(10) $$ROS=\;\frac{Operating\;Profit}{Sales}$$

(11) $$ROI=\;\frac{Earnings\;after\;taxation}{Total\;Asset-Short-term\;resources}$$

(12) $$LRR=\;\frac{Wages\;and\;Salaries}{Total\;Sales}$$

(13) $$WAR=\;\frac{Wages\;and\;Salaries}{Added\;Value}$$

(14) $$LP=\;\frac{Added\;Value}{Number\;of\;Employees}$$

(15) $$DA=\;\frac{Liabilities+Acruals}{Assets}$$

(16) $$DE=\;\frac{Total\;Debt}{Equity}$$

(17) $$FL=\;\frac{Assets}{Equity}$$

(18) $$DIR=\;\frac{Assets}{Liabilities}$$

(19) $$DCR=\;\frac{Profit}{Interst+Principal}$$

(20) $$ACR=\;\frac{Equity+Liabilities}{Fixed\;Assets}$$

(21) $$BL=\;\frac{Bank\;Liabilities\;}{Total\;Debt}$$
